# Real‐World Outcomes of Acute Myeloid Leukemia Patients Undergoing Treatment With Tyrosine Kinase Inhibitor (TKI) Therapy Targeting FLT3, IDH1, or IDH2

**DOI:** 10.1002/cnr2.70657

**Published:** 2026-08-16

**Authors:** Manuel R. Espinoza‐Gutarra, Brooke Jarret, Xiaoliang Wang, Anosheh Afghahi, Sejong Bae

**Affiliations:** ^1^ The University of Alabama at Birmingham O'Neal Comprehensive Cancer Center Birmingham Alabama USA; ^2^ Flatiron Health New York New York USA; ^3^ University of Colorado School of Medicine Aurora Colorado USA; ^4^ Wellstar MCG Health Medical Center Augusta Georgia USA; ^5^ Augusta University Georgia Cancer Center Augusta Georgia USA

**Keywords:** acute myeloid leukemia, AML, real world evidence, targeted therapy

## Abstract

**Background and Aims:**

Acute myeloid leukemia (AML) remains the most common cause of death in adults with acute leukemia. Tyrosine kinase inhibitors (TKIs) have shown significant clinical efficacy in defined patient subgroups. Real‐world outcomes of AML patients undergoing TKI therapy have not been widely reported.

**Methods:**

In this real‐world retrospective study, we included 482 adult patients with AML treated with a TKI targeting FLT3, IDH1, or IDH2 from January 1, 2015, to December 31, 2023, in the countrywide Flatiron Health electronic health record‐derived deidentified database. We utilized unadjusted Kaplan–Meier methods to calculate median real‐world event‐free survival (rwEFS) and overall survival (rwOS). We utilized a multivariable Cox regression model to evaluate characteristics associated with rwOS, modeling post‐TKI stem cell transplant (SCT) as a time‐varying covariate.

**Results:**

Those who received treatment at 1L (first‐line) and 2L+ (second‐line and beyond) had similar length of TKI therapy (3.7 vs. 3.4 months), rwEFS (2.2 vs. 2.5 months), and rwOS (12.3 vs. 13.1 months), respectively. Among all patients, greater rwOS was associated with receiving pre‐TKI SCT (*p* < 0.001), commercial insurance (vs. Medicaid; *p* = 0.035), younger age (*p* = 0.043), and favorable 2017 ELN risk classification (vs. adverse; *p* = 0.032). When restricted to patients treated with TKI at 2L+ without prior SCT, rwEFS were 1.4, 1.6, and 2.1 months by targetable mutation (IDH2, IDH1, and FLT3), and rwOS were 8.7, 15.4, and 9.5 months, respectively.

**Conclusion:**

Patients treated with HMA plus TKI did not have significant differences compared to TKI alone. In this substantial, real‐world cohort of AML patients receiving TKIs, clinical outcomes remained deficient irrespective of timing of TKI start, mutational target, and concurrent HMA.

## 1 Introduction

Acute myeloid leukemia (AML) is the predominant adult acute leukemia in the United States with an incidence rate of 3–5 per 100 000, and it accounts for 3%–4% of all cancer deaths [1]. Despite high mortality, improvements in clinical outcomes such as event‐free survival (EFS), overall survival (OS), and quality of life (QoL) among others have been gradual, mostly stemming from the introduction of hypomethylating agents (HMA) and venetoclax combination therapy. Recent advancements in newly FDA‐approved targeted therapies, like FLT3, IDH1, and IDH2 inhibitors, have so far shown promise for improving patients' overall and event‐free survival in the setting of clinical trials [2].

The identification and targeting of these mutations is one of the most important advancements in AML therapeutics in the last decade; for instance, FLT3 is present in about 20%–25% of AML patients in both its tyrosine kinase domain (TKD) and in‐tandem duplication (ITD) variants. This mutation, particularly in its ITD variant, is known to carry significantly worse OS and EFS, and its targeting—in combination with intensive chemotherapy, less intense treatment, and in the post‐SCT setting—has led to improvements in remission rates and survival [3]. IDH1 and IDH2 mutations are present in 7%–10% and 10%–20% of patients with AML; these mutations have traditionally been associated with intermediate prognosis. Interestingly, both are known to be very highly sensitive to venetoclax‐based therapy, and there are currently trials evaluating the utility of targeted therapy, either in combination with chemotherapy or in the post‐SCT setting, currently ongoing [4].

Tyrosine kinase inhibitors (TKIs) like Gilteritinib (to target FLT3), Ivosidenib and Olutasidenib (to target IDH1), and Enasidenib (to target IDH2) have recently shown some promise in achieving complete remission (CR) and composite complete remission (CRc). Gilteritinib was the first FLT3 inhibitor to be approved as monotherapy by the FDA for treatment of relapsed/refractory (R/R) AML. Early phase data showed CR and CRc rates of 11% and 41%, respectively, which were then confirmed in a Phase 3 trial showing rates of 21% and 54 (vs. 13% and 27% in the placebo control arm) [5, 6, 7]. Ivosidenib was approved to treat R/R IDH1 mutant AML based on Phase 1/2 data showing CR and CRc rates of 27% and 38% [8]. A recent Phase 3 trial also evaluated ivosidenib in combination with azacitidine (vs. azacitidine alone) and showed improved rates of CRc (47% vs. 15%) [9]. Olutasidenib is a newly developed IDH1 inhibitor which was also approved based on Phase 1/2 data showing OS rates of 8.7 months as monotherapy and 12.1 months in combination with azacitidine [10]. Enasidenib has also been approved for R/R IDH2 mutant AML, where it showed CR and CRc rates of 20% and 27%, respectively [11] but its EFS and OS (4.9 and 6.5 months respectively) did not differ significantly from control arm [12], while a retrospective multicenter study also significantly lower rates of CRc for 2L Enasidenib when compared with other therapies (16.9% vs. 26.3%) [13]. A recent trial in combination with azacitidine showed a 70% CRc and 39% CR rates, respectively [14]. OS and EFS estimates for these targeted therapies remain low, with most generally outperforming control arms but not exceeding 2 years.

Currently available guidelines, like from the NCCN, suggest the use of TKI as first line in combination with intensive induction chemotherapy only for FLT3 mutant patients. However, in patients who are not candidates for intensive induction, guidelines recommend ivosidenib in combination with azacitidine for IDH1 mutant patients; other combinations, including TKI monotherapy, are recommended as second or third line options for IDH2 and FLT3 mutant patients [15]. Additionally, the European Leukemia Net (ELN) 2024 guidelines for patients not eligible for intensive chemotherapy consider IDH1 and IDH2 mutant patients as favorable risk and FLT3 mutated patients as intermediate risk, based on results from above mentioned clinical trials wherein patients have been treated either by TKI ± HMA or venetoclax‐based combinations [16].

Despite the promising results from these clinical trials, there is little evidence of the clinical outcomes among patients receiving these targeted therapies in the real‐world setting. Previous studies have shown that clinical outcomes, including OS and EFS, of clinical trial patients far outperform those in the real world, in other cancers such as lung cancer [17] and multiple myeloma [18]. This difference, however, has not been evaluated in the context of targeted TKI therapy for AML patients. Our group previously compared real‐world outcomes of TKI therapy between racial/ethnic minority patients and non‐Hispanic White (NHW) patients. The aforementioned study was explicitly designed to assess health [19]. In contrast, the present study is the first to use the Flatiron database to evaluate TKI‐treated patients as a whole in a thorough fashion, by reporting clinical results across multiple target mutations (IDH1, IDH2, EFT3) and comparing monotherapy to combination therapy (with the addition of HMA). Specifically, in this study, we aim to describe the real‐world event‐free survival (rwEFS) and overall survival (rwOS) in a sizable cohort of AML patients treated with TKIs, as well as to describe factors associated with improved outcomes and its results will highlight the real‐world impact of these widely used therapeutics regardless of race or ethnicity.

## 2 Methods

### 2.1 Study Population

This analysis used the nationwide Flatiron Health electronic health record (EHR)‐derived deidentified database, consisting of deidentified, patient‐level structured and unstructured data systematized via technology‐enabled abstraction [20]. Throughout the timespan of the study, the deidentified data stemmed from around 280 US cancer clinics (~800 sites of care). The majority of patients in the dataset stemmed from community oncology settings; comparative academic/community ratios change based on the disease group analyzed. This study was developed and executed under research agreement between Flatiron Health and collaborating institutions. Ad Hoc Institutional Board Review Approval was not required for this study given its use of summarized and unidentified data only. Given the deidentified nature of the dataset, informed consent requirement was waived.

Patients were incorporated in the analysis if they had a verified AML diagnosis (excluding acute promyelocytic leukemia and mixed phenotype leukemia), were ≥ 18 years at time of diagnosis, and were confirmed to receive a TKI targeting FLT3, IDH1, or IDH2 as monotherapy or in combination with a hypomethylating agent from January 1, 2015, until December 31, 2023. Patient demographics, clinical characteristics, AML‐related information, and therapeutics were obtained via structured and unstructured documents in the EHR. The ELN 2017 risk classification [21] was ascertained from available cytogenetic and molecular data. We used the same dataset as noted in the prior publication by our group, which focused on health disparities across different ethnicities; however, we are currently describing clinical outcomes for the main cohort of AML patients [19].

### 2.2 Exposure Groups

Exposure groups were defined by the line in which a patient received their first targeted therapy to account for patients possibly receiving multiple targeted therapies. Lines of therapy (LOTs) in the study were oncologist‐defined and rule‐based. We grouped patients into first TKI usage in either first‐line (1L) or second‐line and beyond (2L+).

### 2.3 Clinical Outcomes

The primary results of this study encompassed rwEFS and rwOS. For rwEFS, our study deemed any of the following to be an event: induction failure, relapse, or death. Using ELN [21], IWG [22], and FDA [23] guidelines for response assessment in AML clinical trials and routine clinical practice, we considered patients to have had an induction failure or relapse if they had any of the following documented in their chart:

- A bone marrow blast percentage of > 5% as captured from unstructured EHR data, specifically lab reports, pathology reports, and clinician notes;
- A provider assessment of therapeutic effect based on bone marrow aspirate/biopsy results (including blast percentages, Auer rod evidence, presence of extramedullary disease, etc.) equivalent to “Failure to Respond” (e.g., patient has induction failure, residual or persistent disease, partial or incomplete response) or “Relapse” (e.g., patient's AML has relapsed or recurred) done within 28 days of the aspirate/biopsy; and/or
- A peripheral blast percentage of > 0% as recorded in the aforementioned unstructured EHR data or structured lab data using LOINCs corresponding to “Blasts/100 leukocytes in Blood” (e.g., 26446‐5). This threshold (> 0%) is conservative as compared to some registries like CIBMTR, which uses > 5%.

Evidence of *any* induction failure or relapse from these sources constituted an event, even if the sources were discrepant. The definition above focused on clinical, lab, and pathological evidence and did not count LOT advancements as “events.” Events may be undercaptured if they were documented outside of the data sources listed above (e.g., extramedullary disease was discovered only in radiographic evidence).

For rwOS, the Flatiron Health database ascertains death status by using data from EHR complemented with commercially available sources and United States Social Security Death Index data and has been benchmarked against the National Death Index [24]. In order to maintain deidentification, date of death was only obtainable at the month‐level and/or year‐level granularity in the underlying data.

### 2.4 Statistical Analyses

We performed a descriptive analysis of patients' baseline clinical and treatment‐related characteristics, and by LOT 1L versus 2L+. For continuous variables, we estimated medians and interquartile ranges (IQRs). For categorical variables, we estimated frequencies and percentages.

We performed multiple time‐to‐event analyses using treatment initiation as the index date. We estimated the median duration of TKIs, defined as the time from the first drug episode to the last drug episode of the targeted therapy (alone or in combination with an HMA) within the index line. We used unadjusted Kaplan–Meier product limit estimator methods to compare median rwEFS and rwOS by LOT (1L vs. 2L+). For rwEFS inquiry, patients were censored at the last date recorded during which a patient had the potential to be observed to have an event (i.e., the last time that a clinic note was recorded, a laboratory specimen was obtained, or a peripheral blast was observed). For rwOS analyses, patients were censored at last confirmed patient activity (i.e., the latest measurement of patients' vital signs, medication delivery, laboratory testing, or abstracted therapeutic data). A two‐sided log‐rank test was utilized to assess statistically significant differences at an *α* level of 0.05. Resultant *p* < 0.05 were deemed “statistically significant.”

We utilized univariable and multivariable Cox regression models to evaluate variables associated with rwOS. For the Cox multivariable model, we selected covariates a priori based on a directed acyclic graph and clinical expertise to ensure the inclusion of relevant confounders. Covariates included age at index, sex assigned at birth, race/ethnicity, year of the index date, practice type, ECOG score, blast percentage, the LOT where TKI was used (1L, 2L, 3L, or 4L+), insurance status, ELN risk stratification, SCT before TKI start, and, as a time‐varying covariate, SCT after TKI start. We calculated the events per variable (~13) and checked all variable inflation factors (all < 2.5) to reduce overfitting and reduce predictor redundancy. We addressed missing covariate values using multiple (five) imputations by chained equations for missing values of race/ethnicity, ECOG, and blast percentage.

After observing the results from the aforementioned analyses, we decided to examine only patients who received TKI in 2L+ without prior SCT in a post hoc fashion. This was done to both exclude patients who receive TKI as post‐SCT maintenance, which is associated with high OS and EFS rates, and to generate a cohort that better resembles published clinical trial data where patients are mostly treated in the R/R setting. Then, we evaluated rwEFS and rwOS by targetable mutation and compared TKI monotherapy versus TKI + HMA doublet therapy.

Statistical analyses were performed using R 4.1.3 [25]. Survival estimates, Kaplan–Meier curves, and cox proportional hazards models were implemented via survminer [26] and survival packages [27]. To handle tied survival times, we used an Efron approximation. We tested the proportional hazards assumption for all covariates using Schoenfeld residuals quantitatively and a visual inspection of plots; we assessed the linearity of continuous variables (e.g., age at index) with Martingale residuals. There were no substantial violations.

## 3 Results

In aggregate, 482 patients were included in the analysis; baseline clinical and treatment related characteristics are noted on Table 1. Median age at diagnosis was 69 (IQR: 60, 76) while median age at TKI initiation was 70 (IQR: 61, 77). Males accounted for 52% of patients, 70% were NHW, Medicare was the primary insurance for 48.1% of patients, 52% received treatment in a community setting (versus an academic medical center), 18.4% received TKI + HMA, 52% of patients had an ECOG of 0–1, and 10% of patients had SCT after initiating TKI treatment. Per ELN 2017, 42% were classified as adverse, 52% as intermediate, and 6% as favorable; additionally, 4.4% had extramedullary disease at diagnosis.

**TABLE 1:** Baseline demographic and clinical data.

	Overall, *N* = 482	1L, *n* = 68	2L+, *n* = 414
Age at targeted therapy initiation			
Median (IQR)	70 (61, 77)	77 (72, 83)	68 (60, 75)
Age at diagnosis			
Median (IQR)	69 (60, 76)	77 (72, 83)	67 (58, 74)
Sex			
Female	231 (48%)	32 (47%)	199 (48%)
Male	251 (52%)	36 (53%)	215 (52%)
Race and ethnicity			
Non‐Hispanic White	335 (70%)	42 (62%)	293 (71%)
Hispanic or Latino (any race)	27 (5.6%)	< 5^a^	> 5
Black (non‐Hispanic or unknown ethnicity)	27 (5.6%)	< 5	> 5^a^
Asian (non‐Hispanic or unknown ethnicity)	16 (3.3%)	< 5^a^	> 5^a^
A race not listed above (non‐Hispanic or unknown ethnicity)	39 (8.1%)	7 (10%)	32 (7.7%)
Unknown/not documented	38 (7.9%)	8 (12%)	30 (7.2%)
Practice type			
Community	249 (52%)	45 (66%)	204 (49%)
Academics	233 (48%)	23 (34%)	210 (51%)
ECOG performance score at targeted therapy initiation			
0	89 (18%)	14 (21%)	75 (18%)
1	162 (34%)	23 (34%)	139 (34%)
2+	76 (16%)	13 (19%)	63 (15%)
Unknown	155 (32%)	18 (26%)	137 (33%)
AML diagnosis preceded by MDS or MPN			
Yes	151 (31%)	34 (50%)	117 (28%)
No/unknown	331 (69%)	34 (50%)	297 (72%)
Extramedullary disease present at diagnosis			
Yes	21 (4.4%)	< 5^a^	> 5^a^
No/unknown	461 (96%)	> 5^a^	> 5^a^
Treatment‐related AML			
Yes	23 (4.8%)	5 (7.4%)	18 (4.3%)
No/unknown	459 (95%)	63 (93%)	396 (96%)
Insurance category			
Commercial Health Plan	132 (27%)	11 (16%)	121 (29%)
Medicaid	19 (3.9%)	< 5^a^	> 5^a^
Medicare	232 (48.1%)	46 (68%)	186 (45%)
Other payers	36 (7.5%)	< 5^a^	> 5^a^
Uninsured/unknown	63 (13%)	6 (8.8%)	57 (14%)
European LeukemiaNet (ELN) 2017 risk classification			
Adverse	201 (42%)	29 (43%)	172 (42%)
Intermediate	252 (52%)	> 5^a^	> 5
Favorable	29 (6.0%)	< 5^a^	> 5^a^
Stem cell transplant with respect to therapy initiation			
Transplant ONLY before therapy initiation, or before and after therapy initiation	132 (27%)	< 5^a^	> 5^a^
Transplant ONLY after therapy initiation	49 (10%)	< 5^a^	> 5
No transplant before nor after therapy initiation	301 (62%)	64 (94%)	237 (57%)
Index regimen type			
Gilteritinib alone	179 (37%)	13 (19%)	166 (40%)
Gilteritinib + HMA	30 (6.2%)	< 5^a^	> 5
Ivosidenib alone	68 (14%)	12 (18%)	56 (14%)
Ivosidenib + HMA	38 (7.9%)	17 (25%)	21 (5.1%)
Olutasidenib alone	< 5^a^	< 5^a^	< 5^a^
Olutasidenib + HMA	< 5^a^	< 5^a^	< 5^a^
Enasidenib alone	142 (29%)	16 (24%)	126 (30%)
Enasidenib + HMA	21 (4.4%)	7 (10%)	14 (3.4%)
First targeted therapy received			
Monotherapy	395 (81.6%)	42 (61.8%)	353 (84.9%)
With HMA	89 (18.4%)	26 (38.2%)	63 (15.1%)

Abbreviations: 1L: first‐line; 2L+: second‐line and beyond.

^a^ Cells with fewer than five patients are masked as < 5 for privacy; other cells that would allow for back‐calculation are also masked.

**FIGURE 1:**
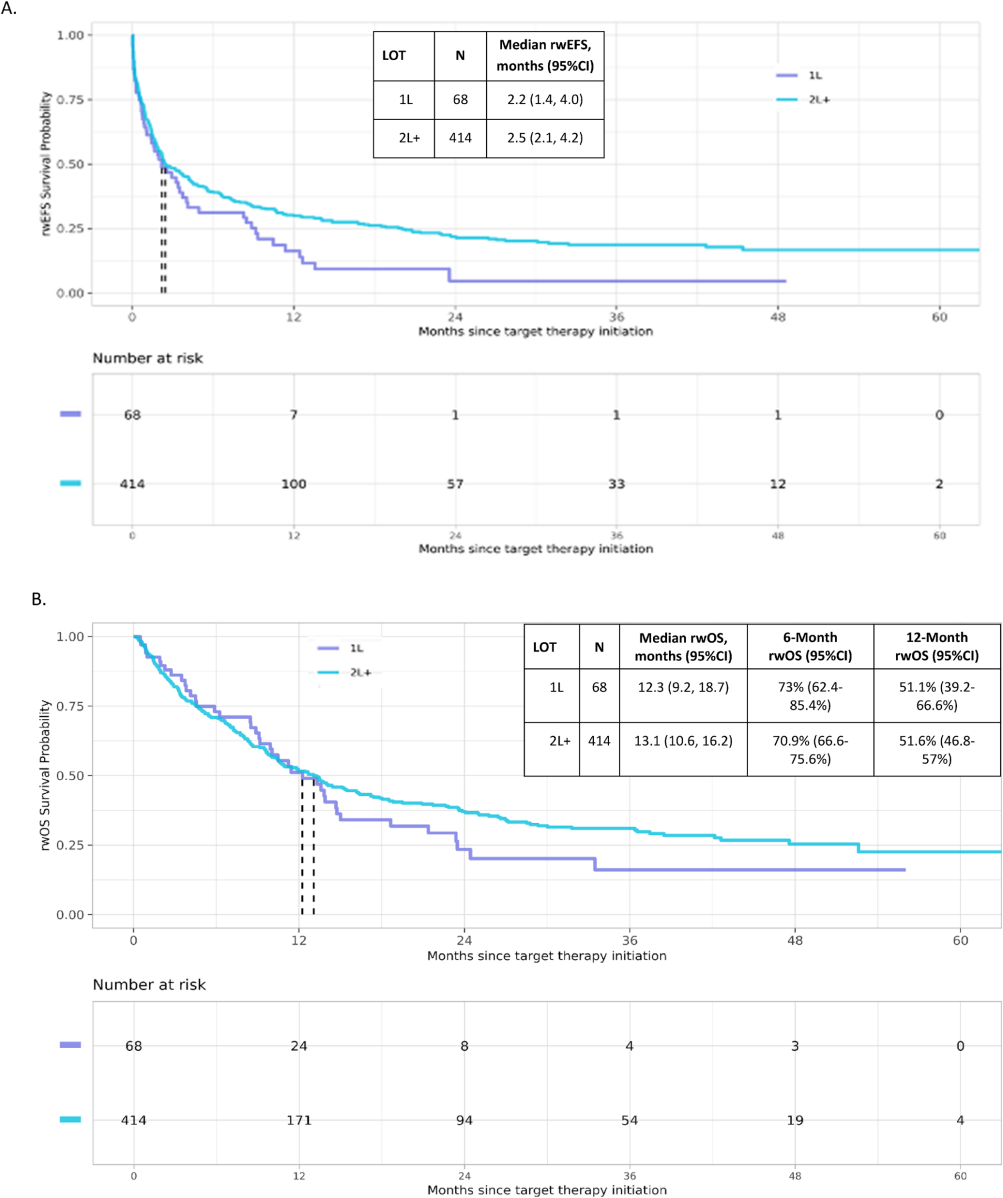
Unadjusted Kaplan–Meier curves describing rwEFS and rwOS among AML patients (excluding promyelocytic and mixed phenotype leukemia) who were ≥ 18 years at diagnosis and treated with a TKI targeting FLT3, IDH1, or IDH2 as monotherapy or with an HMA from January 1, 2015, until December 31, 2023 from Flatiron Health Electronic Health Record (EHR)‐derived data. Index date is date of TKI initiation. Censor dates were defined as the last encounter with potential for event capture (e.g., notes, labs) for rwEFS and the last confirmed clinical activity (e.g., vitals, treatment, or lab dates) for rwOS. 1L: first‐line; 2L+: second‐line and beyond. (A) rwEFS and (B) rwOS.

TKIs were administered as 1L therapy for 14% (*n* = 68) of patients and 86% (*n* = 414) in second‐line or beyond (2L+). Median age at TKI initiation was 77 years for 1L patients, 62% identified as NHW, 15% had commercial health insurance, and 50% had prior myelodysplastic syndromes (MDSs) or myeloproliferative neoplasm (MPN). Patients treated with a TKI at 2L+ had a median age at index of 68 years, 71% were NHW, 29% had commercial health insurance, and 28% had prior MDS or MPN. Patients treated at 1L and 2L+ had a comparable median duration of therapy (3.7 vs. 3.4 months), rwEFS (2.2 [95% confidence interval [CI] 1.4–4.0] vs. 2.5 [95% CI: 2.1–4.2] months), and rwOS (12.3 [95% CI: 9.2–18.7] vs. 13.1 [95% CI: 10.6–16.2] months). Additionally, landmark 6 and 12‐month rwOS were 73.0% [95% CI: 62.4%–85.4%] and 51.1% [95% CI: 39.2%–66.6%] for 1L and 70.9% [95% CI: 66.6%–75.6%] and 51.6% [95% CI: 46.8%–57.0%] for 2L+, respectively (Figure 1a,b).

The adjusted multivariable analysis is presented in Table 2. Among all patients, an increased hazard of death was statistically significantly associated with older age at TKI initiation (*p* = 0.0194; adjusted hazard ratio [aHR] = 1.02 [1.00–1.03] per year older) and Medicaid (vs. commercial insurance; *p* = 0.029; aHR = 2.07 [1.08–3.98]). Conversely, a significantly reduced hazard of death was associated with pre‐TKI SCT (*p* < 0.001; aHR = 0.43 [0.30–0.62]) and favorable 2017 ELN risk (vs. adverse; *p* = 0.032; aHR = 0.52 [0.29–0.94]). Receiving a TKI in 1L was associated with better rwOS when compared to later lines (2L, 3L, 4L+), albeit only the 3L versus 1L (*p* = 0.012; HR = 1.67 [1.12–2.49]) and 4L+ versus 1L (*p* = 0.007; HR = 1.83 [1.18–2.83]) comparisons were statistically significant, showing an increased hazard of death for patients who started in TKI in a later line. Receiving SCT post‐TKI reduced the adjusted hazard of death by 23% [95% CI: 0.54–1.41] but this was not statistically significant (*p* = 0.573), perhaps due to low overall sample size number (*n* = 49).

**TABLE 2:** Multivariate analysis of factors associated with improved rwOS.

	Unadjusted (*n* = 482^a^)			Adjusted (*n* = 482)^b^		
	HR	95% CI	*p*	HR	95% CI	*p*
Age at index (continuous)	1.03	1.02–1.04	**< 0.001** *	1.02	1.00–1.03	**0.019** *
Female	—	—	—	—	—	—
Male	1.14	0.91–1.44	0.266	1.15	0.91–1.46	0.251
Non‐Hispanic (NH) White	—	—	—	—	—	—
Hispanic/Latino and Black, Asian, or race not previously listed who are NH or ethnicity unknown^c^	1.15	0.87–1.52	0.318	0.91	0.67–1.22	0.514
Year of index date (continuous)	0.95	0.88–1.03	0.243	0.96	0.89–1.04	0.331
ECOG 0	—	—	—	—	—	—
1	0.96	0.68–1.36	0.828	0.92	0.64–1.33	0.654
2+	1.57	1.07–2.32	**0.022** *	1.17	0.78–1.76	0.442
Unknown	0.88	0.62–1.25	0.473	—	—	—
Blast percentage (≤ 20%)	—	—	—	—	—	—
> 20%	0.92	0.64–1.33	0.674	0.99	0.68–1.45	0.960
Unknown/not documented	0.89	0.51–1.57	0.697	—	—	—
Targeted therapy in 1L (*n* = 68)	—	—	—	—	—	—
Targeted therapy in 2L (*n* = 191)	0.83	0.58–1.18	0.299	1.16	0.79–1.68	0.446
Targeted therapy in 3L (*n* = 130)	0.94	0.64–1.37	0.735	1.67	1.12–2.49	**0.012** *
Targeted therapy in 4L+ (*n* = 93)	0.84	0.57–1.26	0.401	1.83	1.18–2.83	**0.007** *
Insurance (REF: Commercial Health Plan)	—	—	—	—	—	—
Medicaid	1.64	0.89–3.03	0.113	2.07	1.08–3.98	**0.029** *
Medicare	1.57	1.18–2.09	**0.002** *	1.25	0.92–1.70	0.157
Other payers	1.57	0.98–2.51	0.063	1.75	1.07–2.86	**0.027** *
Uninsured/unknown	0.92	0.61–1.41	0.714	1.14	0.73–1.79	0.558
ELN risk category (REF: adverse)	—	—	—	—	—	—
Intermediate	0.92	0.73–1.17	0.491	0.92	0.72–1.17	0.485
Favorable	0.57	0.32–1.01	0.054	0.52	0.29–0.94	**0.032** *
Transplant before TKI start (REF: no/unknown)	—	—	—	—	—	—
Yes	0.40	0.30–0.54	**< 0.001** *	0.43	0.30–0.62	**< 0.001** *
Transplant after TKI (REF: no)	—	—	—	—	—	—
Yes	0.77	0.5–1.19	0.237	0.87	0.54–1.41	0.573

^a^ Sample size was *n* = 482 for univariate and multivariable models unless otherwise noted. Multiple imputation by chained equations was used with five imputations for missing values of race/ethnicity, blast percentage, and ECOG in the multivariable model.

^b^ Multivariable model also adjusted for the type of practice where care was received (community oncology group vs. academic medical center). However, Flatiron does not recommend presenting results on survival outcomes by practice type due to differences in documentation and data integration.

^c^ *n* = 444 in this univariate model due to race/ethnicity missing for 38 patients.

* Statistically significant results (*p* < 0.05) are bolded.

**FIGURE 2:**
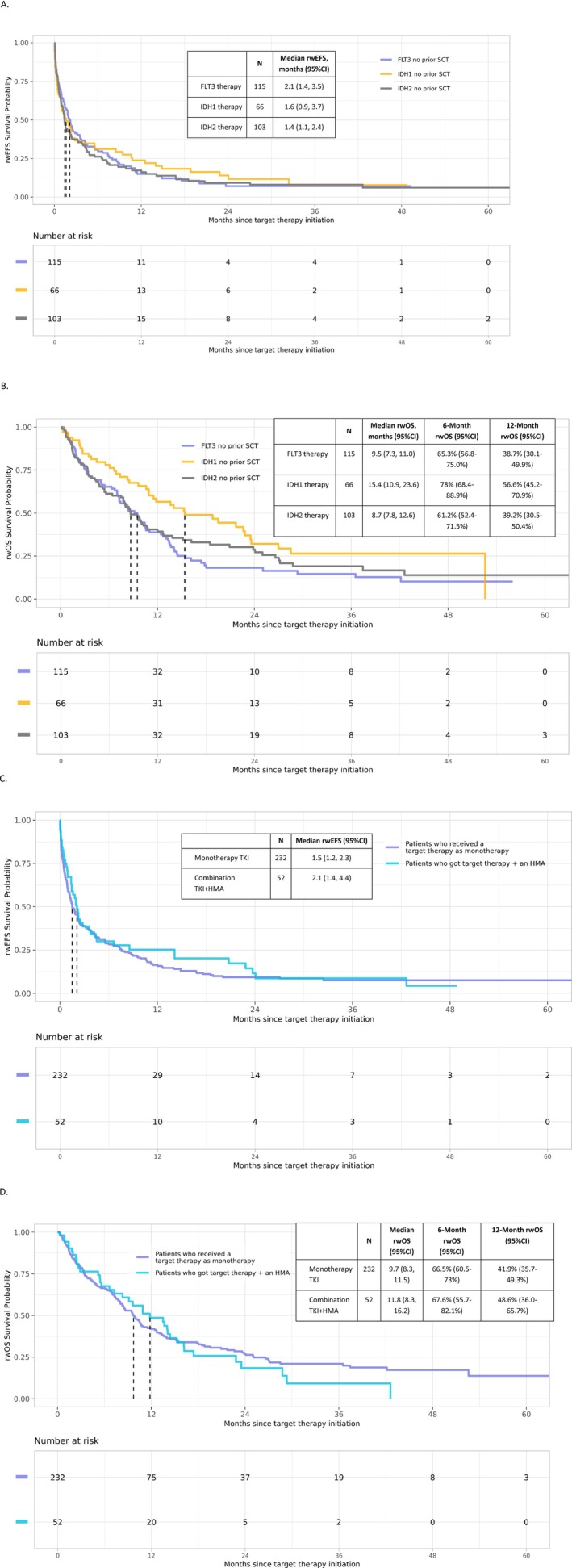
Unadjusted Kaplan–Meier curves describing rwEFS and rwOS among AML patients (excluding promyelocytic and mixed phenotype leukemia) who were ≥ 18 years at diagnosis, did not receive prior SCT and were treated with a TKI targeting FLT3, IDH1, or IDH2 in 2L+ as monotherapy or with an HMA from January 1, 2015, until December 31, 2023, from Flatiron Health Electronic Health Record (EHR)‐derived data. Index date is date of TKI initiation. Censor dates were defined as the last encounter with potential for event capture (e.g., notes, labs) for rwEFS and the last confirmed clinical activity (e.g., vitals, treatment, or lab dates) for rwOS. (A) rwEFS by mutation. (B) rwOS by mutation. (C) rwEFS TKI monotherapy versus combination therapy (TKI + HMA). (D) rwOS TKI monotherapy versus combination therapy (TKI + HMA).

When analysis was limited to patients who were treated with TKI at 2L+ without prior SCT, rwEFS was 2.1 [95% CI: 1.4–3.5], 1.6 [95% CI: 0.9–3.7], and 1.4 [95% CI: 1.1–2.4] months by target (FLT3, IDH1, and IDH2) respectively, and rwOS was 9.5 [95% CI: 7.3–11.0], 15.4 [95% CI: 10.9–23.6], and 8.7 [95% CI: 7.8–12.6] months respectively. When evaluating rwOS by landmark dates, the 6 and 12‐month rwOS were 65.3% [95% CI: 56.8%–75.0%] and 38.7% [95% CI: 30.1%–49.9%] for FLT3; 78.0% [95% CI: 68.4%–88.9%] and 56.6% [95% CI: 45.2%–70.9%] for IDH1; and 61.2% [95% CI: 52.4%–71.5%] and 39.2% [95% CI: 30.5%–50.4%] for IDH2, respectively (Figure 2a,b). Patients who were treated with TKI + HMA did not have statistically significant differences compared to monotherapy in rwEFS (2.1 [95% CI: 1.4–4.4] vs. 1.5 [95% CI: 1.2–2.3] months) or rwOS (11.8 [95% CI: 8.3, 16.2] vs. 9.7 [95% CI: 8.3, 11.5]); with landmark 6 and 12‐month rwOS of 66.5% [95% CI: 60.5%–73.0%] and 41.9% [95% CI: 35.7%–49.3%] for TKI monotherapy and 67.6% [95% CI: 55.7%–82.1%] and 48.6% [95% CI: 36.0%–65.7%] for TKI + HMA (Figure 2c,d).

## 4 Discussion

In this real‐world analysis of treatment for AML patients receiving therapy with TKIs targeting FLT3, IDH1, and IDH2, we observed that rwEFS and rwOS remain limited regardless of treatment setting.

Across all analyzed patients, improved rwOS was associated with younger age, having SCT prior to TKI treatment, having commercial insurance (vs. Medicaid), favorable 2017 ELN risk (vs. adverse), and first line TKI therapy (vs. third or fourth line). Age is a recognized prognostic factor in AML and a predictor of treatment response; however, most of these data come from patients undergoing chemotherapy‐based treatment [28]; our data supports that this relationship is maintained in the TKI era. SCT continues to show its importance in achieving long‐term remissions and improvement in OS, which has been shown in post hoc analysis of clinical trials [7, 29] and small retrospective series [30]. Regarding insurance, prior reports have shown that being uninsured or on Medicaid [31] or on Medicare [32] were associated with inferior OS; in our study only Medicare insurance was associated with inferior rwOS in univariable analysis, and Medicaid and other payers were the only insurance associated with inferior rwOS in multivariable analysis. Bade et al. has attributed decreased OS of Medicare beneficiaries to lower rates of molecular profiling in this population [32]. However, our study population selected only for those who already had molecular testing, which suggests there are other causes. Favorable ELN 2017 risk status was also associated with better rwOS in our study. In other studies evaluating older patients, ELN 2017 risk stratification failed to be predictive of survival outcomes, even when patients were treated with intensive chemotherapy [33, 34], additionally, a study evaluating the fitness of ELN 2022 in older patients treated with either chemotherapy, HMA, or HMA plus venetoclax also showed the lack of accuracy of these risk models [35]; it is interesting that in our analysis ELN 2017 did maintain some predictive value, which could be due to FLT3 mutation being represented across all risk strata and that this represents a larger sample of mostly older patients treated with TKIs. Finally, while our results show that treating patients with TKIs in first line was associated with better rwOS when compared to third and fourth line, this is likely a reflection of subsequent therapies prolonging survival in patients treated with TKIs in first line, particularly, given the increased number of FDA‐approved treatments now available. A current ongoing trial is evaluating the benefits of first versus second line targeted therapy in IDH1/2 mutated AML patients who are unsuitable for intensive chemotherapy [36].

We then evaluated clinical outcomes of patients who received TKIs in 2L+ and did not receive an SCT before TKI initiation. This was done post hoc after noting large differences between patients receiving TKIs in 1L versus 2L+ and to exclude any patients in whom TKI might have been given as post‐SCT maintenance. Although we did not conduct formal analyses to compare this study's results with clinical trial data, we draw some qualitative comparisons here regarding differences by mutational target.

Regarding FLT3, using gilteritinib median EFS (mEFS) was 2.8 months in the Phase 3 trial [5] and was not reported in the early phase trial [6], this compares favorably with 2.1 months rwEFS in our cohort. In those same trials, median OS (mOS) was 7 months in the early phase and 9.3 months in Phase 3, which is very similar to our rwOS of 9.5 months. In IDH1 patients, treated with ivosidenib, median duration of response (mDoR), which evaluates time‐to‐event in patients who have at least achieved a partial response and correlates positively with EFS, was 8.2 months and mOS was 8.8 months in a Phase 1/2 trial [8], this mOS increased to 12.6 months in a subset analysis of newly diagnosed (ND) patients only [37], which compares to the rwEFS of 1.6 months and rwOS of 15.4 months in our analysis, again showing the potential effect of subsequent therapies improving OS in our real‐world cohort. We had < 5 patients treated with olutasidenib, which makes comparisons with existing data difficult for this subset of patients. In IDH2 patients, enasidenib showed a mDOR and mEFS of 5.8 and 4.9 months in early [11] and Phase 3 trials [12], respectively, and mOS of 9.3 and 6.5 months in the same trials, this compares favorably with our analysis showing rwEFS of 1.4 months and rwOS of 8.7 months. Interestingly, another large real‐world retrospective study reported PFS and OS of 8 and 11 months, respectively, however, this was restricted to 2L patients only and its PFS definition only accounted for overt progression or death, which might explained difference in findings, additionally, landmark OS at 6 and 12 months were 80% and 42%, respectively, which still exceeds our reported rwOS of 61.2% and 39.2% at those same timepoints [13].

Across all AML patients treated with TKI monotherapy, our analysis shows that EFS is longer in clinical trials when compared to our real‐world database, however, OS did not show any major differences, the difference in EFS is likely explained by clinical trial patients generally being more fit, more compliant and with less comorbidities than real‐world patients which can lead to lower rates of toxicity and treatment failure and higher adherence to treatment; and the similar OS values are likely driven by availability of additional treatments, particularly HMA and venetoclax, the latter of which was not available during the aforementioned trials likely due to venetoclax being FDA approved for use in AML patients on November 2018 [38], when most of these trials had completed accrual. Additionally, both IDH1 and IDH2 mutant AML are known to be very responsive to HMA plus venetoclax combination therapy and are considered as favorable risk in the 2024 ELN classification of AML patients receiving less‐intensive therapies [16]. Smaller retrospective reports on the interaction between TKI monotherapy and HMA plus venetoclax have shown that when venetoclax is used as salvage, mOS is around 9.2 months [39] and when TKIs are used as salvage, mOS are 3.9 [40] and 4.2 [41] months from initiation of targeted therapies, which are more consistent with the results of this study.

When evaluating HMA plus TKI combination therapy we found that rwEFS was numerically lower than what has been reported in registrational trials, while rwOS remained somewhat similar. A Phase 3 trial evaluating gilteritinib plus azacitidine in FLT3 mutant ND AML showed a mEFS of 4.53 months and mOS of 9.82 months, which did not improve outcomes when compared to the azacitidine monotherapy comparator arm [42]. For IDH1, the combination of azacitidine and ivosidenib was initially studied in a Phase 1b trial in ND AML patients with a median duration of treatment (mDOT) of 15 months and a mOS that was not reached at 16 months of follow‐up, but an 82% survival rate at 12 months [43]; these encouraging results were replicated in a Phase 3 trial evaluating the same combination, showing an initial mOS of 24 months and a 1‐year EFS probability of 37% [9], with longer follow‐up of this same trial showing a mOS of 29.3 months at a median follow‐up of 28.6 months [44]; interestingly, a large real‐world analysis by Smith et al. evaluating ivosidenib plus HMA versus HMA plus venetoclax in IDH1 mutant patients showed a 6 month EFS of 56% versus 39.6% when considering CR within 24 weeks as part of the definition [45]. For IDH2, the azacitidine plus enasidenib combination in ND AML showed a mEFS of 15.9 months and mOS of 22 months in a Phase 1b/2 trial [46].

Our analysis showed a rwEFS of 2.1 months and rwOS of 11.8 months across patients receiving HMA plus TKI combination in the 2nd line without a prior SCT, which continues to show the shorter pattern of rwEFS in real‐world cohorts, likely a result of patient selection and the different definitions of EFS used in clinical trials [22] when compared to real‐world studies. Interestingly, this difference was less notable when evaluating rwOS; this is likely due to the role of post‐progression and salvage therapies which are able to improve OS despite a shorter EFS, and are particularly effective in IDH1/2 mutant patients [47]. Finally, IDH1 mutant patients had a numerically greater rwOS, which is consistent with this population being particularly sensitive to less‐intensive therapies. Additionally, another study published by our group evaluating the same Flatiron cohort of AML patients focused on disparities in clinical outcomes including rwEFS and rwOS in NHW patients versus patients of color found that the latter were more likely to receive treatment in a community hospital, have Medicaid as primary insurance, and to be in the lowest socioeconomic index quintile when compared to NHW; however this did not translate in differences in rwEFS and rwOS either in the 1L or 2L+ settings [19].

In conclusion, our analysis represents the largest study—to our knowledge—to evaluate real‐world outcomes of AML patients undergoing TKI therapy for targetable mutations. It demonstrated that well‐known risk factors affect survival, and rwEFS results are consistently numerically lower than those noted in clinical trials. Real‐world data, along with confirmatory Phase 3 studies shine a light on the actual clinical utility of targeted therapies that receive conditional approval based on response rates in early phase trials. One such example is enasidenib monotherapy, which was removed from the Canadian market after the confirmatory Phase 3 trial failed to show improvement over best available care [48], and while patient‐level trial analysis has shown that response rates and EFS are associated with OS [49], this relationship remains to be replicated in large, real‐world post‐marketing studies. In addition to this study our group recently published an analysis evaluating the impact of TKI in AML patients of diverse racial/ethnic origin, finding a rwEFS of 1.5 and 2 months and a rwOS of 9.7 and 9.9 months for NHW and POC (patients of color) respectively, these findings complement our current study, showing that TKI use and effectiveness might ameliorate health disparities in AML therapy, while we used the same dataset for this analysis, as noted in the methods section, the current study has a wider scope, evaluating outcomes for the entire cohort and by LOT, TKI target and use of HMA, which are all novel findings in relation to the mentioned companion piece [19].

There are several limitations in our study, which include lack of data availability in our cohort, which precludes us from analyzing response rates, including CR and CRi, our inability to use more recent ELN risk stratification models, general data missingness, lack of sample size to analyze specific subgroups separately (e.g., IDH1 mutant AML treated with olutasidenib or HMA + TKI treated patients stratified by specific mutation), and inability to differentiate intention of TKI therapy (e.g., post‐SCT maintenance versus first line or salvage therapy) which could potentially affect the results from the primary cohort, however, it should be noted that the rwEFS and rwOS of the full cohort and of the 2L+ TKI without prior SCT were similar, indicating a likely minor impact of possible post‐SCT maintenance patients in this study. Additionally, while a missing indicator method maintains sample size, we acknowledge that this approach may introduce bias due to non‐random missingness like different documentation strategies across practice settings. We aimed to mitigate some of this risk by increasing event capture through both structured and unstructured data. With new approvals coming to the market every year, questions of timing and sequencing [50] of these agents remain open, but the very significant decline in both rwEFS and rwOS curves early on and the overall low number of patients able to reach the 24‐month mark signals the continued dire need for effective therapies in this patient population.

## Author Contributions

**Xiaoliang Wang:** formal analysis, data curation, writing – review and editing, supervision. **Brooke Jarret:** methodology, validation, writing – review and editing, supervision, data curation, formal analysis. **Sejong Bae:** writing – review and editing, investigation, supervision, data curation, formal analysis. **Manuel R. Espinoza‐Gutarra:** conceptualization, investigation, writing – original draft, writing – review and editing. **Anosheh Afghahi:** writing – review and editing, visualization, supervision.

## Funding

The authors have nothing to report.

## Ethics Statement

This study received an exempt determination from the institutional review board (IRB) of the O'Neal Comprehensive Cancer Center at UAB and execute under Flatiron and UAB CDA.

## Conflicts of Interest

Manuel R. Espinoza‐Gutarra reports consulting fees: Abbvie, Stemline, Incyte, Gamida Cell. Research support: BMS Foundation. Speaking fees: AAMDSIF, NMDP. The other authors declare no conflicts of interest.

## Data Availability

The data that support the findings of this study are available from Flatiron Health Inc. Restrictions apply to the availability of these data, which were used under license for this study. Data are available from the author(s) with the permission of Flatiron Health Inc.

## References

[1] R. L. Siegel , T. B. Kratzer , A. N. Giaquinto , H. Sung , and A. Jemal , “Cancer Statistics, 2025,” CA: A Cancer Journal for Clinicians 75, no. 1 (2025): 10–45. 39817679 10.3322/caac.21871 PMC11745215

[2] J. Wang , B. Tomlinson , and H. M. Lazarus , “Update on Small Molecule Targeted Therapies for Acute Myeloid Leukemia,” Current Treatment Options in Oncology 24, no. 7 (2023): 770–801. 37195589 10.1007/s11864-023-01090-3

[3] N. Daver , R. F. Schlenk , N. H. Russell , and M. J. Levis , “Targeting FLT3 Mutations in AML: Review of Current Knowledge and Evidence,” Leukemia 33, no. 2 (2019): 299–312. 30651634 10.1038/s41375-018-0357-9 PMC6365380

[4] G. C. Issa and C. D. Dinardo , “Acute Myeloid Leukemia With IDH1 and IDH2 Mutations: 2021 Treatment Algorithm,” Blood Cancer Journal 11, no. 6 (2021): 107. 34083508 10.1038/s41408-021-00497-1 PMC8175383

[5] A. E. Perl , G. Martinelli , J. E. Cortes , et al., “Gilteritinib or Chemotherapy for Relapsed or Refractory FLT3−Mutated AML,” New England Journal of Medicine 381, no. 18 (2019): 1728–1740. 31665578 10.1056/NEJMoa1902688

[6] A. E. Perl , J. K. Altman , J. Cortes , et al., “Selective Inhibition of FLT3 by Gilteritinib in Relapsed or Refractory Acute Myeloid Leukaemia: A Multicentre, First‐In‐Human, Open‐Label, Phase 1‐2 Study,” Lancet Oncology 18, no. 8 (2017): 1061–1075. 28645776 10.1016/S1470-2045(17)30416-3 PMC5572576

[7] A. E. Perl , R. A. Larson , N. A. Podoltsev , et al., “Outcomes in Patients With FLT3‐Mutated Relapsed/ Refractory Acute Myelogenous Leukemia Who Underwent Transplantation in the Phase 3 ADMIRAL Trial of Gilteritinib Versus Salvage Chemotherapy,” Transplantation and Cellular Therapy 29, no. 4 (2023): 265.e1–265.e10. 10.1016/j.jtct.2022.12.006 PMC10189888 36526260

[8] C. D. Dinardo , E. M. Stein , S. Botton , et al., “Durable Remissions With Ivosidenib inIDH1−Mutated Relapsed or Refractory AML,” New England Journal of Medicine 378, no. 25 (2018): 2386–2398. 29860938 10.1056/NEJMoa1716984

[9] P. Montesinos , C. Recher , S. Vives , et al., “Ivosidenib and Azacitidine inIDH1−Mutated Acute Myeloid Leukemia,” New England Journal of Medicine 386, no. 16 (2022): 1519–1531. 35443108 10.1056/NEJMoa2117344

[10] J. M. Watts , M. R. Baer , J. Yang , et al., “Olutasidenib Alone or With Azacitidine in IDH1‐Mutated Acute Myeloid Leukaemia and Myelodysplastic Syndrome: Phase 1 Results of a Phase 1/2 Trial,” Lancet Haematology 10, no. 1 (2023): e46–e58. 36370742 10.1016/S2352-3026(22)00292-7 PMC12250719

[11] E. M. Stein , C. D. DiNardo , D. A. Pollyea , et al., “Enasidenib in Mutant IDH2 Relapsed or Refractory Acute Myeloid Leukemia,” Blood 130, no. 6 (2017): 722–731. 28588020 10.1182/blood-2017-04-779405 PMC5572791

[12] S. de Botton , P. Montesinos , A. C. Schuh , et al., “Enasidenib vs Conventional Care in Older Patients With Late‐Stage Mutant‐IDH2 Relapsed/Refractory AML: A Randomized Phase 3 Trial,” Blood 141, no. 2 (2023): 156–167. 35714312 10.1182/blood.2021014901 PMC10644040

[13] A. J. Klink , A. Gajra , R. L. Knoth , et al., “Real‐World Clinical Outcomes With Enasidenib in Relapsed or Refractory Acute Myeloid Leukemia,” Leukemia Research 122 (2022): 106946. 36108427 10.1016/j.leukres.2022.106946

[14] S. Venugopal , K. Takahashi , N. Daver , et al., “Efficacy and Safety of Enasidenib and Azacitidine Combination in Patients With IDH2 Mutated Acute Myeloid Leukemia and Not Eligible for Intensive Chemotherapy,” Blood Cancer Journal 12, no. 1 (2022): 1–7. 35078972 10.1038/s41408-021-00604-2 PMC8789767

[15] D. A. Pollyea , J. K. Altman , R. Assi , et al., “Acute Myeloid Leukemia, Version 3.2023, NCCN Clinical Practice Guidelines in Oncology,” Journal of the National Comprehensive Cancer Network 21, no. 5 (2023): 503–513. 37156478 10.6004/jnccn.2023.0025

[16] H. Döhner , C. D. DiNardo , F. R. Appelbaum , et al., “Genetic Risk Classification for Adults With AML Receiving Less‐Intensive Therapies: The 2024 ELN Recommendations,” Blood 144, no. 21 (2024): 2169–2173. 39133932 10.1182/blood.2024025409

[17] S. Peters , R. J. B. Salomonsen , I. Diaz Perez , et al., “Real‐World Outcomes Versus Clinical Trial Results of First‐Line Immunotherapy With or Without Chemotherapy in Patients With Metastatic Non‐Small‐Cell Lung Cancer: The CORRELATE Study,” ESMO Open 11, no. 1 (2026): 106031. 41494223 10.1016/j.esmoop.2025.106031 PMC12810545

[18] A. Visram , K. K. Chan , H. Seow , et al., “Comparing the Clinical Trial Efficacy Versus Real‐World Effectiveness of Treatments for Multiple Myeloma: A Population‐Based Study,” Haematologica 110, no. 1 (2025): 228–233. 39219457 10.3324/haematol.2024.285768 PMC11694101

[19] M. R. Espinoza‐Gutarra , B. A. Jarrett , X. Wang , A. Afghahi , and S. Bae , “Health Disparities in Acute Myeloid Leukemia Patients Undergoing Treatment With Tyrosine Kinase Inhibitor (TKI) Therapy Targeting FLT3, IDH1, or IDH2,” Blood and Lymphatic Cancer 16 (2026): 559759. 41847630 10.2147/BLCTT.S559759 PMC12990797

[20] X. Ma , L. Long , S. Moon , A. BJS , and S. S. Baxi , Comparison of Population Characteristics in Real‐World Clinical Oncology Databases in the US: Flatiron Health, SEER, and NPCR (Cold Spring Harbor Laboratory., 2020).

[21] H. Döhner , E. Estey , D. Grimwade , et al., “Diagnosis and Management of AML in Adults: 2017 ELN Recommendations From an International Expert Panel,” Blood 129, no. 4 (2017): 424–447. 27895058 10.1182/blood-2016-08-733196 PMC5291965

[22] B. D. Cheson , J. M. Bennett , K. J. Kopecky , et al., “Revised Recommendations of the International Working Group for Diagnosis, Standardization of Response Criteria, Treatment Outcomes, and Reporting Standards for Therapeutic Trials in Acute Myeloid Leukemia,” Journal of Clinical Oncology 21, no. 24 (2003): 4642–4649. 14673054 10.1200/JCO.2003.04.036

[23] FDA, Oncology Center of Excellence, Center for Drug Evaluation and Research, Center for Biologics Evaluation and Research , “Acute Myeloid Leukemia: Developing Drugs and Biological Products for Treatment Guidance for Industry,” 2022, cited November 2, 2026, FDA‐2020‐D‐1298, https://www.fda.gov/regulatory‐information/search‐fda‐guidance‐documents/acute‐myeloid‐leukemia‐developing‐drugs‐and‐biological‐products‐treatment‐0.

[24] Q. Zhang , A. Gossai , S. Monroe , N. C. Nussbaum , and C. M. Parrinello , “Validation Analysis of a Composite Real‐World Mortality Endpoint for Patients With Cancer in the United States,” Health Services Research 56, no. 6 (2021): 1281–1287. 33998685 10.1111/1475-6773.13669 PMC8586476

[25] R Development Core Team , R: A Language and Environment for Statistical Computing (R Foundation for Statistical Computing, 2026).

[26] A. Kassambara , M. Kosinski , P. Biecek , and S. Fabian , survminer: Drawing Survival Curves using ‘ggplot2’: R Package Version 0.4.9 (The R Foundation for Statistical Computing, 2021).

[27] P. Grambsch and T. M. Therneau , Modeling Survival Data: Extending the Cox Model (Springer, 2020).

[28] B. Oran and D. J. Weisdorf , “Survival for Older Patients With Acute Myeloid Leukemia: A Population‐Based Study,” Haematologica 97, no. 12 (2012): 1916–1924. 22773600 10.3324/haematol.2012.066100 PMC3590098

[29] C. D. Dinardo , E. M. Stein , A. Pigneux , et al., “Outcomes of Patients With IDH1‐Mutant Relapsed or Refractory Acute Myeloid Leukemia Receiving Ivosidenib Who Proceeded to Hematopoietic Stem Cell Transplant,” Leukemia 35, no. 11 (2021): 3278–3281. 33772143 10.1038/s41375-021-01229-x PMC8464620

[30] A. Genthon , D. Dragoi , M. Memoli , et al., “Isocitrate Dehydrogenase Inhibitors as a Bridge to Allogeneic Stem Cell Transplant in Relapsed or Refractory Acute Myeloid Leukaemia,” British Journal of Haematology 198, no. 4 (2022): 780–784. 35615877 10.1111/bjh.18290

[31] D. Pulte , F. A. Castro , H. Brenner , and L. Jansen , “Outcome Disparities by Insurance Type for Patients With Acute Myeloblastic Leukemia,” Leukemia Research 56 (2017): 75–81. 28212899 10.1016/j.leukres.2017.02.001

[32] R. Bade , L. G. Banaszak , F. Osman , et al., “Neighborhood Disadvantage, Insurance Status, and Molecular Profiling of Patients With Acute Myeloid Leukemia,” Leukemia Research 131 (2023): 107326. 37263074 10.1016/j.leukres.2023.107326

[33] R. Itzykson , E. Fournier , C. Berthon , et al., “Genetic Identification of Patients With AML Older Than 60 Years Achieving Long‐Term Survival With Intensive Chemotherapy,” Blood 138, no. 7 (2021): 507–519. 34410352 10.1182/blood.2021011103

[34] E. L. Pogosova‐Agadjanyan , A. Moseley , M. Othus , et al., “AML Risk Stratification Models Utilizing ELN‐2017 Guidelines and Additional Prognostic Factors: A SWOG Report,” Biomarker Research 8, no. 1 (2020): 29. 32817791 10.1186/s40364-020-00208-1 PMC7425159

[35] S. Park , T. Y. Kim , B.‐S. Cho , et al., “Prognostic Value of European Leukemia Net 2022 Criteria and Genomic Clusters Using Machine Learning in Older Adults With Acute Myeloid Leukemia,” Haematologica 109, no. 4 (2024): 1095–1106. 37706344 10.3324/haematol.2023.283606 PMC10985444

[36] M. P. Ozga , K. M. Dvorak‐Kornaus , Q. Zhao , et al., “I‐DATA Study: Randomized, Sequential, Open‐Label Study to Evaluate the Efficacy of IDH Targeted/Non‐ Targeted Versus Non‐Targeted/IDH‐Targeted Approaches in the Treatment of Newly Diagnosed IDH Mutated Adult AML Patients Not Candidates for Intensive Induction Therapy,” Blood 142, no. Supplement 1 (2023): 1534.

[37] G. J. Roboz , C. D. DiNardo , E. M. Stein , et al., “Ivosidenib Induces Deep Durable Remissions in Patients With Newly Diagnosed IDH1‐Mutant Acute Myeloid Leukemia,” Blood 135, no. 7 (2020): 463–471. 31841594 10.1182/blood.2019002140 PMC7019193

[38] FDA , “FDA Approves Venetoclax in Combination for AML in Adults,” 2018, January 18 2025, https://www.fda.gov/drugs/fda‐approves‐venetoclax‐combination‐aml‐adults.

[39] J. P. Bewersdorf , R. M. Shallis , A. Derkach , et al., “Venetoclax‐Based Salvage Therapy in Patients With Relapsed/Refractory Acute Myeloid Leukemia Previously Treated With FLT3 or IDH1/2 Inhibitors,” Leukemia and Lymphoma 64, no. 1 (2023): 188–196. 36287540 10.1080/10428194.2022.2136952 PMC9905301

[40] V. Khanna , T. Azenkot , S. ( Q.) Liu , et al., “Outcomes With Molecularly Targeted Agents as Salvage Therapy Following Frontline Venetoclax + Hypomethylating Agent in Adults With Acute Myeloid Leukemia: A Multicenter Retrospective Analysis,” Leukemia Research 131 (2023): 107331. 37263072 10.1016/j.leukres.2023.107331

[41] J. P. Bewersdorf , R. M. Shallis , A. Derkach , et al., “Efficacy of FLT3 and IDH1/2 Inhibitors in Patients With Acute Myeloid Leukemia Previously Treated With Venetoclax,” Leukemia Research 122 (2022): 106942. 36108424 10.1016/j.leukres.2022.106942

[42] E. S. Wang , P. Montesinos , M. D. Minden , et al., “Phase 3 Trial of Gilteritinib Plus Azacitidine vs Azacitidine for Newly Diagnosed FLT3mut+ AML Ineligible for Intensive Chemotherapy,” Blood 140, no. 17 (2022): 1845–1857. 35917453 10.1182/blood.2021014586 PMC10653009

[43] C. D. Dinardo , A. S. Stein , E. M. Stein , et al., “Mutant Isocitrate Dehydrogenase 1 Inhibitor Ivosidenib in Combination With Azacitidine for Newly Diagnosed Acute Myeloid Leukemia,” Journal of Clinical Oncology 39, no. 1 (2021): 57–65. 33119479 10.1200/JCO.20.01632 PMC7771719

[44] S. D. Botton , S. de Botton , P. Montesinos , et al., “Updated Efficacy and Safety Data From the AGILE Study in Patients With Newly Diagnosed Acute Myeloid Leukemia Treated With Ivosidenib + Azacitidine Compared to Placebo + Azacitidine,” Journal of Clinical Oncology 41, no. 16_suppl (2023): 7012.

[45] B. D. Smith , C. A. Lachowiez , A. J. Ambinder , et al., “A Comparison of Acute Myeloid Leukemia (AML) Regimens: Hypomethylating Agents Combined With Ivosidenib or Venetoclax in Newly Diagnosed Patients With IDH1 Mutations: A Real‐World Evidence Study,” Blood 142, no. Supplement 1 (2023): 971.

[46] C. D. Dinardo , A. C. Schuh , E. M. Stein , et al., “Enasidenib Plus Azacitidine Versus Azacitidine Alone in Patients With Newly Diagnosed, Mutant‐IDH2 Acute Myeloid Leukaemia (AG221‐AML‐005): A Single‐Arm, Phase 1b and Randomised, Phase 2 Trial,” Lancet Oncology 22, no. 11 (2021): 1597–1608. 34672961 10.1016/S1470-2045(21)00494-0

[47] D. A. Pollyea , C. D. DiNardo , M. L. Arellano , et al., “Impact of Venetoclax and Azacitidine in Treatment‐Naïve Patients With Acute Myeloid Leukemia and IDH1/2 Mutations,” Clinical Cancer Research 28, no. 13 (2022): 2753–2761. 35046058 10.1158/1078-0432.CCR-21-3467 PMC9365354

[48] Goverment of Canada , “IDHIFA (Enasidenib Mesylate)—Market Withdrawal and Continued Access,” January 18, 2025, https://recalls‐rappels.canada.ca/en/alert‐recall/idhifa‐enasidenib‐mesylate‐market‐withdrawal‐and‐continued‐access.

[49] K. J. Norsworthy , X. Gao , C. W. Ko , et al., “Response Rate, Event‐Free Survival, and Overall Survival in Newly Diagnosed Acute Myeloid Leukemia: US Food and Drug Administration Trial‐Level and Patient‐Level Analyses,” Journal of Clinical Oncology 40, no. 8 (2022): 847–854. 34890212 10.1200/JCO.21.01548 PMC8906455

[50] L. Wang , J. Song , X. Xiao , D. Li , T. Liu , and X. He , “Comparison of Venetoclax and Ivosidenib/Enasidenib for Unfit Newly Diagnosed Patients With Acute Myeloid Leukemia and IDH1/2 Mutation: A Network Meta‐Analysis,” Journal of Chemotherapy 36, no. 3 (2024): 202–207. 37599456 10.1080/1120009X.2023.2247200

